# Mangrove sinkholes (*cenotes*) of the Yucatan Peninsula, a global hotspot of carbon sequestration

**DOI:** 10.1098/rsbl.2021.0037

**Published:** 2021-05-05

**Authors:** M. F. Adame, N. S. Santini, O. Torres-Talamante, K. Rogers

**Affiliations:** ^1^ Australian Rivers Institute, Griffith University, Nathan, QLD 4111, Australia; ^2^ Instituto de Ecología, Universidad Nacional Autónoma de México, Mexico City, Mexico; ^3^ Colectividad Razonatura A.C., Mexico City, Mexico; ^4^ University of Wollongong, Wollongong, NSW 2522, Australia

**Keywords:** Caribbean, carbon credits, karst, soil organic carbon, Yucatan, wetlands

## Abstract

Mangroves are among the most carbon-dense ecosystems on the planet. The capacity of mangroves to store and accumulate carbon has been assessed and reported at regional, national and global scales. However, small-scale sampling is still revealing ‘hot spots’ of carbon accumulation. This study reports one of these hotspots, with one of the largest-recorded carbon stocks in mangroves associated with sinkholes (*cenotes*) in the Yucatan Peninsula, Mexico. We assessed soil organic carbon (SOC) stocks, sequestration rates and carbon origin of deep peat soils (1 to 6 m)*.* We found massive amounts of SOC up to 2792 Mg C ha^−1^, the highest value reported in the literature so far. This SOC is primarily derived from highly preserved mangrove roots and has changed little since its deposition, which started over 3220 years ago (±30 BP). Most *cenotes* are owned by Mayan communities and are threatened by increased tourism and the resulting extraction and pollution of groundwater. These hot spots of carbon sequestration, albeit small in area, require adequate protection and could provide valuable financial opportunities through carbon-offsetting mechanisms and other payments for ecosystem services.

## 1. Introduction

Mangroves are among the most carbon-dense ecosystems on the planet and offer significant potential to mitigate atmospheric carbon concentrations [1]. As a result, efforts to map their aerial cover, and associated carbon stocks, have been undertaken through spatial models [2] and intensive field campaigns [3]. However, even the most detailed and accurate global or national model cannot reasonably account for ‘hot spots’ of carbon storage and sequestration, especially when they are very small in area, albeit extremely dense in carbon. Carbon projects are emerging as an innovative conservation tool, where carbon accumulated in ecosystems can be sold through carbon-offset markets [4]. These projects typically need to be large to ensure that the project is financially viable, i.e. that the costs of conservation or restoration are lower than the value of the carbon. However, sites that are very dense in carbon may provide enough credits to meet the project targets. Here, we provide evidence for one of those hotspots in mangroves associated with sinkholes in the Yucatan Peninsula, Mexico.

**Figure 1.**
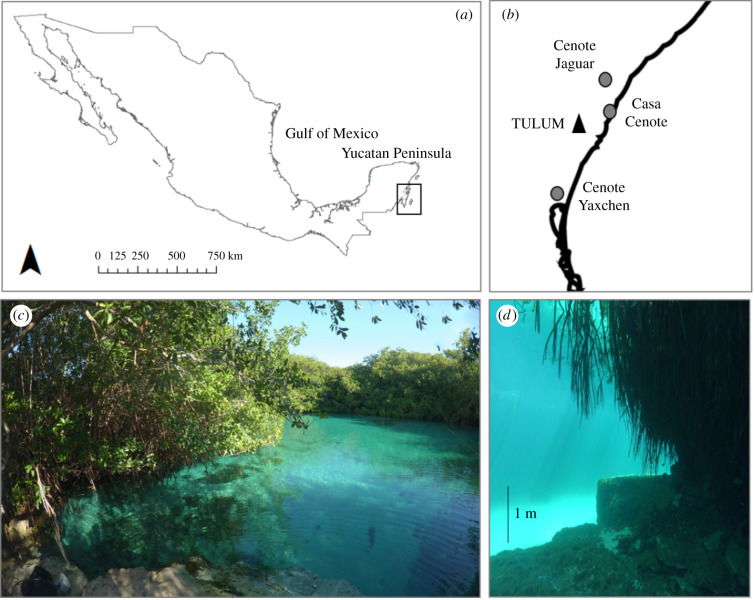
(*a,b*) Sampling sites within the east coast of the Yucatan Peninsula, near Tulum, Mexico; (*c*) the sinkholes are surrounded by dense mangrove forests, (*d*) the peat underneath the mangroves can be 6 m deep (Casa Cenote, pictures MF Adame).

The Yucatan Peninsula is characterized by one of the largest karst aquifer networks in the world, with a surface area of 165 000 km^2^ of permeable limestone [4]. Rainwater slowly dissolves the calcium carbonate forming a groundwater network, with ongoing dissolution eventually leading to the collapse of the limestone [5]. This process can create sinkholes, locally known as *cenotes*, a word derived from the low-land Maya language ‘*tz'onot*’. There are over 2000 *cenotes* distributed throughout the Yucatan Peninsula. In the north, *cenotes* were formed by the impact of the Chicxulub asteroid creating a feature known as ‘Ring of Cenotes’ [6]. In the east, *cenotes* are associated with the Holbox fault, which formed open water bodies parallel to the coastline. In these *cenotes*, seawater intrudes into the aquifer creating a habitat for dense mangrove forests (figure 1). *Cenotes* are highly valuable wetlands that provide habitat for migrating birds and many endemic amphibians and freshwater fish, many of which are classified as endangered [6]. Despite their importance, *cenotes* face significant threats, including groundwater extraction that supports a rapidly growing tourist industry and pollution from agricultural and domestic activities [7].

Preliminary studies have shown that the mangroves associated with groundwater in the Yucatan Peninsula can have very large carbon stocks exceeding 1000 Mg C ha^−1^ [8]. However, these values may underestimate their carbon content due to the difficulty of taking deep soil cores (greater than 2 m). Here, we explored sinkholes surrounded by mangroves in the Yucatan Peninsula, where deep soil samples (up to 6 m) were accessible through underwater caves. The objectives were to obtain carbon stock estimates, identify the origin of this carbon and calculate sequestration rates within mangroves associated with these potential carbon hotspots.

## 2. Methodology

### (a) Study site

Yucatan Peninsula is in the southeast of Mexico. The region has a tropical wet–dry climate (Köppen classification Aw) with a mean annual temperature of 25.7°C (mean annual minimum and maximum of 20.0 and 31.4°C, respectively) and mean annual precipitation of 1122 mm (Tulum Meteorological Station, 1981–2010; [9]). There are three distinct seasons: a cool season of *norte*s (north winds) from November to January with sporadic rains (mean monthly precipitation of 68.3 mm), a hot dry season from March to April (37.7 mm) and a hot wet season from May to October (133.9 mm), when tropical storms are common.

We selected three cenotes associated with mangroves, where SCUBA diving was accessible (figure 1). Casa Cenote (20°16′ N, 87°23.5′ W) is a narrow meandrous cenote located 50 m from the sea with a cave opening of about 1000 m^2^. This cenote has a maximum depth of 8 m, with a halocline at 6.5 m separating brackish (12.5 ppt of salinity) at the surface from marine water at the bottom (35 ppt salinity). Cenote Yaxchen (20°07′52′ N, 87°28′ W) is about 350 m from the sea, with an opening of 6500 m^2^, maximum depth of 7.5 m and a similar halocline profile as Casa Cenote. Finally, Cenote Jaguar (20°19′ N, 87°23′ W) is located furthest from the sea, 2 km inland, with an opening of 500 m^2^, a maximum depth of 8 m and no halocline, with salinity values <3 ppt throughout the water column.

### (b) Methodology

Sampling was conducted in September 2019 with SCUBA equipment. We sampled two points at Cenote Jaguar and Yaxchen and four points at Casa Cenote. Within each point, underwater sampling was conducted with a 60 ml mini core, which resulted in minimum compaction. Sampling was done at each of the following depths: 0–15, 15–30, 30–50, 50–100 cm, and every 100 cm until the bottom of the peat [10], which varied among sites. The deep peats were accessed with the SCUBA equipment from the underwater cave system of these *cenotes* (see video in the electronic supplementary material or https://www.youtube.com/watch?v=lGVYxnlkHu8&feature=youtu.be). In Cenote Jaguar, the peat layer was 1 m deep; in Yaxchen, the peat reached 5 m and in Casa Cenote, it reached 6 m. The samples were refrigerated and transported to the laboratory, where they were oven-dried and weighed. Bulk density was determined from the dry mass per wet volume (expressed as g cm^−3^).

Soil samples were acid-washed with HCl to eliminate carbonates and analysed for soil organic carbon (SOC) and δ^13^C with an elemental analyser coupled with an isotope ratio mass spectrometer [8] (EA-IRMS, Serco System, Griffith University, Brisbane, Australia; analytical standard deviation less than 0.1‰). The stability and origin of soil carbon were determined from the variation of δ^13^C values with depth. We expected that isotope values would remain constant with depth if carbon was stable through time and decomposition was low; on the contrary, isotopic values would increase if decomposition was high [11]. We also expected that if all the carbon were from mangrove roots, δ^13^C would be close to −28‰, a value typical of plants with a C_3_ photosynthetic pathway and common for mangroves in the region [12].

Three soils samples (from 75 cm deep in Cenote Jaguar and 450 cm deep in Casa Cenote and Yaxchen) were radiocarbon dated at Beta Analytics (Miami, USA). The results were corrected for isotopic fractionation and calibrated with the 2013 INTCAL program (Libby half-life, 5568 years) and rounded to the nearest 10 years [13,14]. Carbon sequestration rates were estimated by dividing the SOC stock above the sampled depth by the age of the sample. Comparison between SOC stocks and depth were conducted with a linear regression, where samples were pre-tested for normality and homogeneity of variances with the program SPSS Statistics v26 (IBM, USA).

## 3. Results

The SOC for all sites was 38.5 ± 0.9% (mean ± s.e.), values close to vegetation, indicating that the samples were mostly peat, with little sediment (table 1). Bulk density was low with values of 0.11 ± 0.01 g cm^−3^. The δ^13^C values were remarkably consistent with depth (difference < 2‰ for all sites and depths). Carbon stocks were very high at Casa Cenote and Cenote Yaxchen with a mean value of 1492 ± 549 and 1518 ± 561 Mg C ha^−1^, respectively, with one sampling point in Casa Cenote reaching 2792 Mg C ha^−1^. In comparison, SOC stocks at Cenote Jaguar were moderate with 429 ± 44 Mg C ha^−1^.

**Table 1.** Bulk density (BD; g cm^3^), SOC (%), δ^13^C (‰), SOC stock, radiocarbon age (BP) and sequestration rates (Mg C ha^−1^ yr^−1^) of three mangrove *cenotes* in the Yucatan Peninsula, Mexico; *n* = number of sampling sites. Values are (mean ± s.e.).

depth	BD (g cm^3^)	SOC (%)	δ^13^C (‰)	SOC stock (Mg C ha^−1^)	radiocarbon age (±30 BP)	SOC sequestration (Mg C ha^−1^ yr^−1^)
Cenote Jaguar (*n* = 2)						
0–15	0.10 ± 0.03	41.6 ± 0.1	−28.3	61.7 ± 19.0		
15–30	0.12 ± 0.00	41.4 ± 0.7	−26.8	74.1 ± 0.2		
30–50	0.11 ± 0.01	42.0 ± 0.7	−26.9	94.7 ± 12.4		
50–100	0.09 ± 0.00	43.3 ± 0.7	−27.5	198.2 ± 12.8	2490	
total				428.7 ± 44.4		0.12
Cenote Yaxchen (*n* = 2)						
0–15	0.11 ± 0.05	41.8 ± 0.9	−27.6	66.3 ± 27.0		
15–30	0.09 ± 0.02	40.5 ± 0.2	−27.4	54.7 ± 16.1		
30–50	0.08 ± 0.02	41.5 ± 1.2	−27.9	68.8 ± 14.8		
50–100	0.08 ± 0.01	42.9 ± 0.2	−27.4	180.2 ± 12.0		
100–200	0.09 ± 0.01	40.5 ± 0.4	−27.6	371.5 ± 55.5		
200–300	0.09 ± 0.01	40.7 ± 0.1	−28.6	374.4 ± 33.5		
300–400	0.10	38.7	−27.5	379.7		
400–500	0.10	38.5	−27.9	424.6	3040	
total				1517 ± 561.0		0.61
Casa Cenote (*n* = 4)						
0–15	0.12 ± 0.01	35.6 ± 1.3	−27.5	67.1 ± 6.5		
15–30	0.11 ± 0.01	39.5 ± 0.8	−27.8	67.8 ± 2.8		
30–50	0.13 ± 0.01	36.8 ± 0.7	−27.4	97.0 ± 5.2		
50–100	0.14 ± 0.02	37.1 ± 1.8	−27.8	259.5 ± 44.8		
100–200	0.17 ± 0.05	33.8 ± 3.5	−27.9	537.1 ± 81.8		
200–300	0.12 ± 0.01	33.8 ± 1.1	−28.7	409.6 ± 39.9		
300–400	0.20 ± 0.07	33.8 ± 1.1	−27.9	457.1 ± 41.0		
400–500	0.13	34.4 ± 11.0	−28.1	488.1	3220	
500–600	0.11	38.7	−29.3	426.9		
total				1491 ± 548.6		0.66

Because bulk density and SOC concentration were relatively consistent throughout all sampling sites (table 1), SOC stocks were highly correlated with depth (*R*^2^ = 0.89; *p* = 0.024, *y* = 471.3*x* + 24.8; electronic supplementary material, figure S1). The radiocarbon age of the deep peat samples was 2490 ± 30 BP (75 cm deep), 3040 ± 30 BP and 3220 ± 30 BP (450 cm deep) in Cenote Jaguar, Cenote Yaxchen and Casa Cenote, respectively. Correspondingly, SOC sequestration rates were estimated at 0.12, 0.60 and 0.66 Mg C ha^−1^ yr^−1^ (table 1).

## 4. Discussion

We found massive SOC stocks below mangroves of the *cenotes* close to the sea at Casa Cenote and Cenote Yaxchen, with a mean value of 1504 ± 13 Mg C ha^−1^. The SOC density was higher than stocks reported for other mangroves globally (199–1218 Mg C ha^−1^, means per country, [3]). These SOC stocks were also much higher than those for terrestrial vegetation, which range from 42 Mg C ha^−1^ in deserts to 344 Mg C ha^−1^ in boreal forests, and maximum values in boreal peat soils where SOC stocks can reach 1650 Mg C ha^−1^ [15,16]. Remarkably, one sampling site in Casa Cenote with 2792 Mg C ha^−1^ was the highest SOC stock for mangroves ever recorded (range of 46–2076 Mg C ha^−1^ in 190 plots distributed globally, [3]). The massive SOC from these *cenotes* is a result of high soil carbon content (greater than 38%) and deep soils, which have been preserved for millennia.

**Figure 2.**
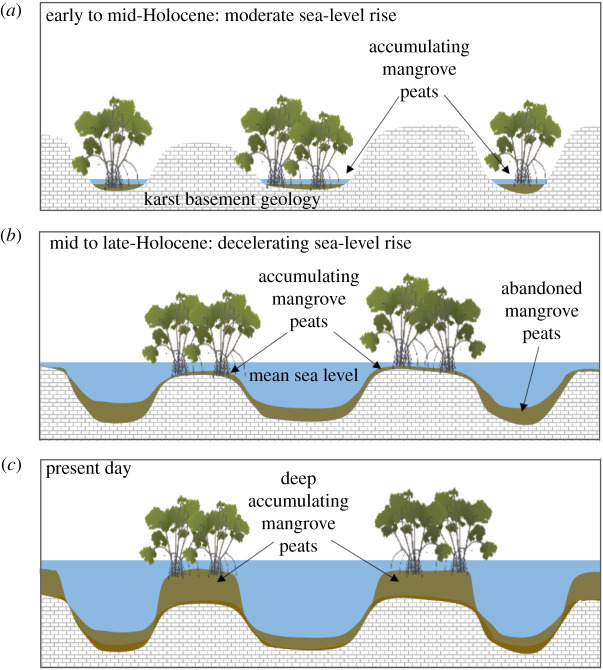
Mangrove peat accumulation in *cenote*s (*a*) during the early to mid-Holocene (11.7–8.2 thousand years BP) when sea level was low but rising at rates beyond the capacity to increase elevation *in situ*, (*b*) during the mid to late-Holocene (8.2–4.2 thousand years BP) when rates of sea-level rise decelerated and mangrove initiated vertical accretion through root production at similar rates to sea-level rise and (*c*) at present day when deep mangrove peats have developed, and vertical growth corresponds to sea-level rise.

We propose that the high SOC stocks of these mangroves are associated with the sea-level history of the region. Globally, after the last glacial minimum, sea-level rise accelerated and then decelerated towards the mid and late-Holocene [17]. At the time of deceleration, conditions were favourable for mangrove expansion [18]. In Yucatan, rates of sea-level rise during the Holocene are uncertain but likely followed a similar pattern [19]. It is possible that during the early to mid-Holocene (11.7–8.2 thousand years BP), the sea level was lower than present levels but rising at rates beyond the capacity of mangroves to increase elevation through root production (figure 2). During the mid- to late-Holocene (8.2–4.2 thousand years BP), sea-level rise decelerated, providing conditions suitable for mangrove vertical growth through root addition and generation of new vertical space, commonly termed ‘accommodation space’, for mangroves to accumulate SOC [20,21]. The age of mangrove peats in this study corresponds to this period of deceleration and suggests SOC accumulation occurred at an average rate of 0.66 Mg C ha^−1^ yr^−1^ in coastal *cenotes* (Cenote Yaxchen and Casa Cenote) and 0.12 Mg C ha^−1^ yr^−1^ in inland *cenote*s (Cenote Jaguar). At present day, deep SOC continues to accumulate, probably at rates similar to those of current sea-level rise [20,21] (figure 2).

The formation of large SOC stocks in these mangroves could also have been favoured by their biological and hydrological characteristics. First, mangroves in the Yucatan Peninsula are highly productive [22,23]; the high root productivity would have allowed for mangroves to accumulate vertically and keep pace with the rising sea [24]. Second, the preservation of accumulated SOC could have been facilitated by increasing submersion over the past millennia due to sea-level rise and the microtidal regime of the region (less than 2 m of tidal amplitude), which allows for these mangroves to be submerged, or at least wet, most of the time. The anoxic conditions caused by submersion limit decomposition and diagenesis and, thus, favour SOC preservation.

The SOC stocks in these *cenotes* are remarkable; however, rates of carbon sequestration are comparable to other mangroves which have lower SOC stocks. For example, SOC sequestration rates in riverine mangroves in southern Mexico are 0.4 to 1.8 Mg C ha^−1^ yr^−1^, but their SOC stocks are lower (less than 700 Mg C ha^−1^ to 1.5 m in depth) [25]. The rates reported in this study represent sequestration occurring over thousands of years synchronous with relative sea-level rise and corresponds to rates of vertical adjustment for other *cenotes* [20] and rates of root production for nearby mangroves (0.64 and 2.6 Mg C ha^−1^ yr^−1^) [23]. While rates of carbon accumulation of other mangroves may be higher, this likely occurs as accommodation space is rapidly infilled with mineral and organic material [24]. Thus, the higher rates of carbon accumulation cannot be sustained over thousands of years as vertical space becomes limited and accumulation increasingly corresponds to rates of relative sea-level rise. Critically, the carbon storage within these mangroves constitutes long-term carbon sequestration as SOC has been stored for thousands of years as evidenced by *δ*^13^C values consistent with a mangrove origin (27–29‰ [12]). Although the additional contribution of terrestrial C_3_ vegetation cannot be discarded, observations of the SOC in the field indicated that it was highly likely to be mangrove roots (see electronic supplementary material video).

The established relationship between SOC stock and depth provides a significant opportunity to reduce costs and effort when estimating SOC for mangroves associated with *cenotes* in this region (electronic supplementary material, figure S1). The massive and stable SOC stocks from this study also provide a strong basis for the inclusion of these mangroves in carbon projects. For instance, a carbon project in a mangrove forest in this region with peat of 2 m would have approximately 1000 Mg C ha^−1,^ from which more than 50% could be lost if the site is converted to another land use [26]. If deforestation rates of the area are 0.1% annually, a conservation project of only 100 ha (the size of the Yaxchen *cenotes* complex) for 100 years could offset almost 13 341 Mg of CO_2eq_ every year [27], which corresponds to over US$13 000 a year within current carbon prices (expecting a value of US$10 per Mg CO_2eq_ in an international market), a considerable amount of money for a rural community in Mexico. This economic incentive could also be complemented through other payments for ecosystem services given the high endemicity of species within these *cenotes* [6] and important cultural values for the Mayan communities, which could be included as part of the Sustainable Development Goals for Mexico.

The exceptionally large SOC stocks stored in these unique ecosystems are threatened by poorly planned tourism, excessive water extraction and groundwater pollution. The latter is caused by the lack of water treating infrastructure in the region, which mostly relies on septic treatments that can leach nitrogen into the aquifer [28]. Contemporary and future sea-level rise would likely increase saltwater intrusion and may favour the expansion of mangroves in new habitats that were not previously inundated. Increased phosphorus in seawater (a key limiting nutrient for these mangroves) could further boost productivity [22,23]. However, urbanization and road construction could significantly impede mangrove inland migration and alter the hydrology of the groundwater network, causing degradation or even massive deaths. The fate of carbon accumulation by existing mangroves is dependent on future rates of sea-level rise, as the threshold of tolerance for vertical adjustment is very likely to be exceeded at rates greater than 5.2 mm yr^−1^ [18]. Thus, the capacity of these mangroves to continue accumulating SOC may become limited at high rates of sea-level rise. Avoiding emissions from these carbon hotspots could be achieved by protecting hydrological networks, avoiding clearing, facilitating inland migration and conducting hydrological restoration in degraded forests including those damaged by tropical storms [29]. These activities could provide economic incentives to the Mayan communities for the sustainable management of *cenote*s within the Yucatan Peninsula.

## Data accessibility

Data are available from the Dryad Digital Repository: https://doi.org/10.5061/dryad.cz8w9gj2x [30].
